# Taxonomic Structure of Rhizosphere Bacterial Communities and Its Association With the Accumulation of Alkaloidal Metabolites in *Sophora flavescens*

**DOI:** 10.3389/fmicb.2021.781316

**Published:** 2021-12-14

**Authors:** Jie Chen, Na Li, Jiayu Chang, Kaida Ren, Jiangtao Zhou, Guan’e Yang

**Affiliations:** School of Pharmaceutical Science, Shanxi Medical University, Taiyuan, China

**Keywords:** medicinal plants, secondary metabolites, alkaloids, plant bacterial community, rhizosphere

## Abstract

Plant secondary metabolites (SMs) play a crucial role in plant defense against pathogens and adaptation to environmental stresses, some of which are produced from medicinal plants and are the material basis of clinical efficacy and vital indicators for quality evaluation of corresponding medicinal materials. The influence of plant microbiota on plant nutrient uptake, production, and stress tolerance has been revealed, but the associations between plant microbiota and the accumulation of SMs in medicinal plants remain largely unknown. Plant SMs can vary among individuals, which could be partly ascribed to the shift in microbial community associated with the plant host. In the present study, we sampled fine roots and rhizosphere soils of *Sophora flavescens* grown in four well-separated cities/counties in China and determined the taxonomic composition of rhizosphere bacterial communities using Illumina 16S amplicon sequencing. In addition, the association of the rhizosphere bacterial microbiota with the accumulation of alkaloids in the roots of *S. flavescens* was analyzed. The results showed that *S. flavescens* hosted distinct bacterial communities in the rhizosphere across geographic locations and plant ages, also indicating that geographic location was a larger source of variation than plant age. Moreover, redundancy analysis revealed that spatial, climatic (mean annual temperature and precipitation), and edaphic factors (pH and available N and P) were the key drivers that shape the rhizosphere bacterial communities. Furthermore, the results of the Mantel test demonstrated that the rhizosphere bacterial microbiota was remarkably correlated with the contents of oxymatrine, sophoridine, and matrine + oxymatrine in roots. Specific taxa belonging to Actinobacteria and Chloroflexi were identified as potential beneficial bacteria associated with the total accumulation of matrine and oxymatrine by a random forest machine learning algorithm. Finally, the structural equation modeling indicated that the Actinobacteria phylum had a direct effect on the total accumulation of matrine and oxymatrine. The present study addresses the association between the rhizosphere bacterial communities and the accumulation of alkaloids in the medicinal plant *S. flavescens*. Our findings may provide a basis for the quality improvement and sustainable utilization of this medicinal plant thorough rhizosphere microbiota manipulation.

## Introduction

Plant secondary metabolites (SMs), produced from secondary metabolic pathways in plants, comprise numerous structurally diverse compounds that can be classified into several large families: phenolics, terpenes, steroids, alkaloids, and flavonoids (Pang et al., 2021). Plant SMs play a crucial role in plant defense against pathogens and pests and adaptation to environmental stresses, also acting as symbiotic signals for plants in association with microbes (Pang et al., 2021). In addition, some plant SMs have been used as ingredients or additives of pharmaceuticals and nutraceuticals, making enormous contributions to public health (Li et al., 2020). However, as a material basis of clinical efficacy and vital indicators for quality evaluation of the corresponding medicinal materials, the SMs of medicinal plants can vary among individuals (Li et al., 2020). Genetic, ontogenetic, and environmental factors are recognized as the sources of variation for the intraspecific polymorphisms of plant SMs (Moore et al., 2014).

Terrestrial plants host taxonomically diverse microorganisms, which exert beneficial, harmful, or neutral effects on plant growth and resistance to biotic and abiotic stresses (Müller et al., 2016). Using next-generation sequencing and bioinformatic analysis, the phylogenetic composition and functional diversities of microbial communities associated with various host plants, such as *Arabidopsis*, several corps, and tree species, have been extensively investigated, revealing associations between plant microbiota and plant nutrient uptake, production, and stress tolerance (Bulgarelli et al., 2012, 2015; Lundberg et al., 2012; Kembel et al., 2014; Edwards et al., 2015; Jin et al., 2017; Zhang et al., 2018a, 2019; Fan et al., 2020; Lee et al., 2021). The community structure and functional capabilities of microbiota associated with medicinal plants have received some research attention recently (Chen et al., 2018; Kui et al., 2021; Liu et al., 2021; Na et al., 2021). It has been proposed that the intraspecific variation in SMs of medicinal plants could be partly ascribed to the shift in the composition of the microbial community associated with the plant host (Huang et al., 2018). However, the associations between plant microbiota and accumulation of SMs in medicinal plants remain largely unknown.

*Sophora flavescens*, a slow-growing Fabaceae shrub, has been planted as an important medicinal plant in China due to its widespread use in pharmacological drugs (Song et al., 2019). The wide-reaching biological activities of SMs of *S. flavescens* mainly include antitumor, antiviral, anti-inflammatory, antianaphylactic, antiarrhythmic, and hepatoprotective effects (He et al., 2015). The root of *S. flavescens* contains extensive bioactive constituents, of which alkaloids are characteristic compounds of the plant and have provoked great interest for their pharmacological effects on cancers, viral hepatitis, and cardiac diseases (He et al., 2015). With the increasing demand for products of medicinal plants and limited arable land, sustainable efforts to improve the production of medicinal plants are required. However, this cannot be achieved by the input of chemical fertilizers and pesticides, which can degrade the quality of medicinal plants (Song et al., 2019). Under these circumstances, the management of favorable microbes is expected to be a potential strategy for facilitating the biosynthesis and accumulation of bioactive components in medicinal plants. Thus, comprehensive studies regarding the association between plant microbiota and bioactive components in medicinal plants are required for the identification of potentially beneficial microbes (Jin et al., 2017).

The plant rhizosphere is a specific microhabitat around the root, where complex soil-microbe-plant interactions occur (Zhang et al., 2017; de Vries et al., 2020). The rhizosphere microbiota, whose genome is referred to as the second genome of plants, contributes to plant phenotype and is vitally important for plant growth and health (Berendsen et al., 2012; Zhang et al., 2017). In addition, the rhizosphere is a hotspot for investigating the interactions between plant-associated microbes and plant SMs (Pang et al., 2021). Therefore, we proposed to address the association between the rhizosphere microbiota and the accumulation of alkaloids in *S. flavescens*.

In the present research, we sequenced the rhizosphere bacterial communities of 72 *S. flavescens* individuals grown in four well-separated areas in China. The key drivers shaping the rhizosphere bacterial communities were evaluated. Moreover, the association of the rhizosphere bacterial microbiota with the accumulation of alkaloids in roots of *S. flavescens* was explored. Potentially beneficial microbes correlated with the total accumulation of matrine and oxymatrine were identified. Finally, the direct and indirect effects of spatial, climatic, and edaphic factors and the preponderant rhizosphere phyla on the accumulation of alkaloids were estimated. We expect that our work will provide a basis for the quality improvement and sustainable utilization of this medicinal plant by rhizosphere microbiota manipulation.

## Materials and Methods

### Sample Collection

Rhizosphere and root samples of *S. flavescens* were collected from 12 fields distributed in four cities/counties in China [Linyuan (LY), Liaoning Province; Changzhi (CZ), Shanxi Province; Dali (DL) and Luonan (LN), Shaanxi Province], which are far-separated with locations between 34 ~ 41° N and 110 ~ 119° E (Supplementary Table S1). The sampling was performed between September and October 2019, roughly corresponding to the period before harvesting. Six individuals from each field were randomly selected as six replicates (*n* = 6). Taking the influence of growth period on rhizosphere microbiota and alkaloid accumulation into consideration, we sampled 1–3-year-old plants (Supplementary Table S1). After shaking off soil particles that were loosely attached, firmly adhering soil layers in combination with roots were collected and transported to our laboratory on ice. In the laboratory, the soil particles firmly attached to roots were gently brushed and collected into sterilized tubes as rhizosphere samples. The rhizosphere soil was stored at −80°C until DNA extraction was performed. The roots were washed with distilled water and oven-dried at 55°C to a constant weight for determination of alkaloid contents.

### DNA Extraction, High-Throughput Sequencing, and Sequence Analysis

Total community DNA was extracted from the rhizosphere samples (0.5 g) using a magnetic soil and stool DNA kit (Tiangen Biotech, Beijing, China) following the manufacturer’s instructions. The V3-V4 hypervariable regions of the 16S rRNA gene were amplified using the specific primers 341F (5′-CCTAYGGGRBGCASCAG-3′) and 806R (5′-GGACTACNNGGGTATCTAAT-3′; Caporaso et al., 2012) with barcodes. Sequencing libraries were generated using the Illumina TruSeq DNA PCR-free library preparation kit (Illumina, San Diego, United States). The library was sequenced on an Illumina NovaSeq platform at Novogene Bioinformatics Technology Co., Ltd. (Beijing, China). Raw reads were processed using the QIIME software package (Caporaso et al., 2010). In detail, the paired-end reads were assigned to each sample based on the unique barcodes, merged, and filtered to obtain high-quality tags. An average of 59,893 qualified reads per sample (ranging from 47,975 to 69,193) was yielded for subsequent analysis. Operational taxonomic units (OTUs) were picked at 97% sequence similarity along with the removal of chimeras (Edgar, 2010; Edgar et al., 2011). Taxonomic assignment was performed against the Silva database (Quast et al., 2013). The nonbacterial OTUs and OTUs whose number of reads was lower than 10 were removed. OTU matrices were rarified to the same depth (42,842 reads per sample). Sequences used in the present research were deposited in the Sequence Read Archive (SRA) dataset of the National Center for Biotechnology Information (NCBI) with the accession number PRJNA780464.

### Acquisition of Soil Physicochemical Parameters and Climatic Data

A subset of the rhizosphere soil was air-dried for determination of its physicochemical properties. Soil pH and the contents of organic matter (OM), available N (AN), available P (AP), and available K (AK) were analyzed following the standard testing procedures described by Bao (2000). Soil pH was measured at 1:1 (soil:deionized water, m/v) ratio using a glass electrode pH meter. The OM content was determined by the potassium dichromate (K_2_Cr_2_O_7_) volumetric method. The AN content was determined by the alkaline hydrolysis diffusion method. AP was extracted with 0.5 M NaHCO_3_ solution and quantified by molybdenum antimony chromatometry. AK was extracted with 1 M NH_4_OAC solution and quantified by flame photometry. The air-dried soil was oven-dried at 105°C to constant weight to determine the moisture content. Data of mean annual temperature (MAT) and mean annual precipitation (MAP) were obtained from the Climatologies at High Resolution for the Earth’s Land Surface Areas (CHELSA).^1^ The soil physiochemical parameters and the climatic data are shown in Supplementary Table S2.

### Determination of Alkaloid Contents in Roots

The oven-dried root samples were ground to a fine powder for the determination of the contents of total alkaloids as well as four alkaloids (oxymatrine, oxysophocarpine, sophoridine, and matrine). Total alkaloids were assayed by the acid dye colorimetry method (Sun et al., 2021) with slight modifications. Briefly, pulverized root samples were ultrasound-treated in 0.2% HCl for 40 min to extract the total alkaloids. The extract was centrifuged, and the supernatant was collected and adjusted to pH 10.0. Subsequently, 5 ml of the supernatant was evaporated to dryness, and the residue was reconstituted in 5 ml of 80% ethanol and thoroughly mixed with 0.2 mM bromothymol blue (pH 7.0) and dichloromethane. Then, the dichloromethane layer was collected for colorimetric assay of the total alkaloids at 417 nm. Oxymatrine, oxysophocarpine, sophoridine, and matrine were analyzed by high-performance liquid chromatography (HPLC; Supplementary Figure S1) according to Pharmacopoeia of the People’s Republic of China (Chinese Pharmacopoeia Commission, 2020). Powdered root samples (0.1 g) were ultrasound-treated in 0.133 ml of 25% ammonia + 8.33 ml of dichloromethane for 40 min for extraction of alkaloids. After the extract was filtered, 5 ml of the filtrate was evaporated, followed by dissolving the residue in methanol for HPLC analysis. Chromatographic separation was achieved on a Shim-pack GIST C18 column (4.6 mm × 250 mm, 5 μm) at 30°C and was detected at an ultraviolet wavelength of 225 nm. A mobile phase consisting of solution A (0.01 M ammonium acetate, pH 8.1) and solution B [acetonitrile: ammonium acetate (0.01 M, pH 8.1), 3:2, v/v] at a flow rate of 1 ml min^−1^ was used. A gradient (A:B, v/v) program of 0 min (9:1) → 20 min (7:3) → 40 min (6:4) → 50 min (4:6) → 60 min (9:1) → 70 min (9:1) was employed. The content of matrine + oxymatrine was calculated. The alkaloid content data are available in Supplementary Table S3.

### Statistical Analysis

The following statistical analyses were performed on the R software platform (R Development Core Team, 2021). The alpha diversity of the bacterial community was calculated for each rhizosphere sample using the species richness and Chao1 and Shannon indices. Significant differences in alpha diversity among the four geographical locations or the three plant ages were determined by Kruskal–Wallis H-tests with the Dunn-Bonferroni *post hoc* method (*P* < 0.05). Spearman’s rank correlation was used to determine the relationships between the Shannon index and environmental variables (including climatic and edaphic variables). Nonmetric multidimensional scaling (NMDS) and principal coordinate analysis (PCoA), both of which were based on the Bray-Curtis distance matrix, were conducted to explore dissimilarities in the rhizosphere bacterial communities and root alkaloid accumulation among samples, respectively. To assess the significance of variation in the rhizosphere bacterial community composition or root alkaloid accumulation across geographical locations or plant ages, permutational multivariate analysis of variance (PERMANOVA) based on Bray–Curtis distances was carried out with 999 permutations. In addition, the significant spatial, climatic, and edaphic variables shaping the rhizosphere bacterial communities were evaluated by redundancy analysis (RDA) with a forward selection procedure identifying the significance (Benjamini-Hochberg adjusted *P* < 0.05). The spatial variables were generated by principal coordinates of neighbor matrices (PCNM) analysis (Borcard et al., 2011; Supplementary Table S2). Based on RDA, variation partitioning analysis (VPA) was performed to measure the relative influence of spatial, climatic, and edaphic factors on the rhizosphere bacterial communities (Peres-Neto et al., 2006).

Random forests and structural equation modeling (SEM) were employed for further analyses. Serving as a robust ensemble machine learning method for classification and regression, a random forest model was applied to identify the specific bacteria associated with the total accumulation of matrine and oxymatrine. The relative abundance of bacterial taxa at the OTU level was regressed against the total content of matrine and oxymatrine by the R package “randomForest” (Zhang et al., 2018c). Ranked lists of bacterial OTUs in order of their feature importance in the random forest model were achieved over 100 iterations of the algorithm. The optimal taxa set correlated with the total content of matrine and oxymatrine was generated based on the minimum errors obtained from 10-fold cross-validation with five repeats. The 30 most important OTUs were chosen to display. The SEM, which allows the assessment of multiple interacting factors and potential causal relationships, was applied to evaluate the direct and indirect effects of spatial (the first axis of principal component analysis for all PCNM vectors), climatic (MAT and MAP), and edaphic (pH, OM, AN, AP, and AK) factors and bacterial attributes (the relative abundance of three dominant rhizosphere phyla) on the accumulation of alkaloids (Fan et al., 2020). The SEM was carried out by using the R package “lavaan.”

## Results

### Diversity and Taxonomic Structure of the Rhizosphere Bacterial Communities

**Figure 1:**
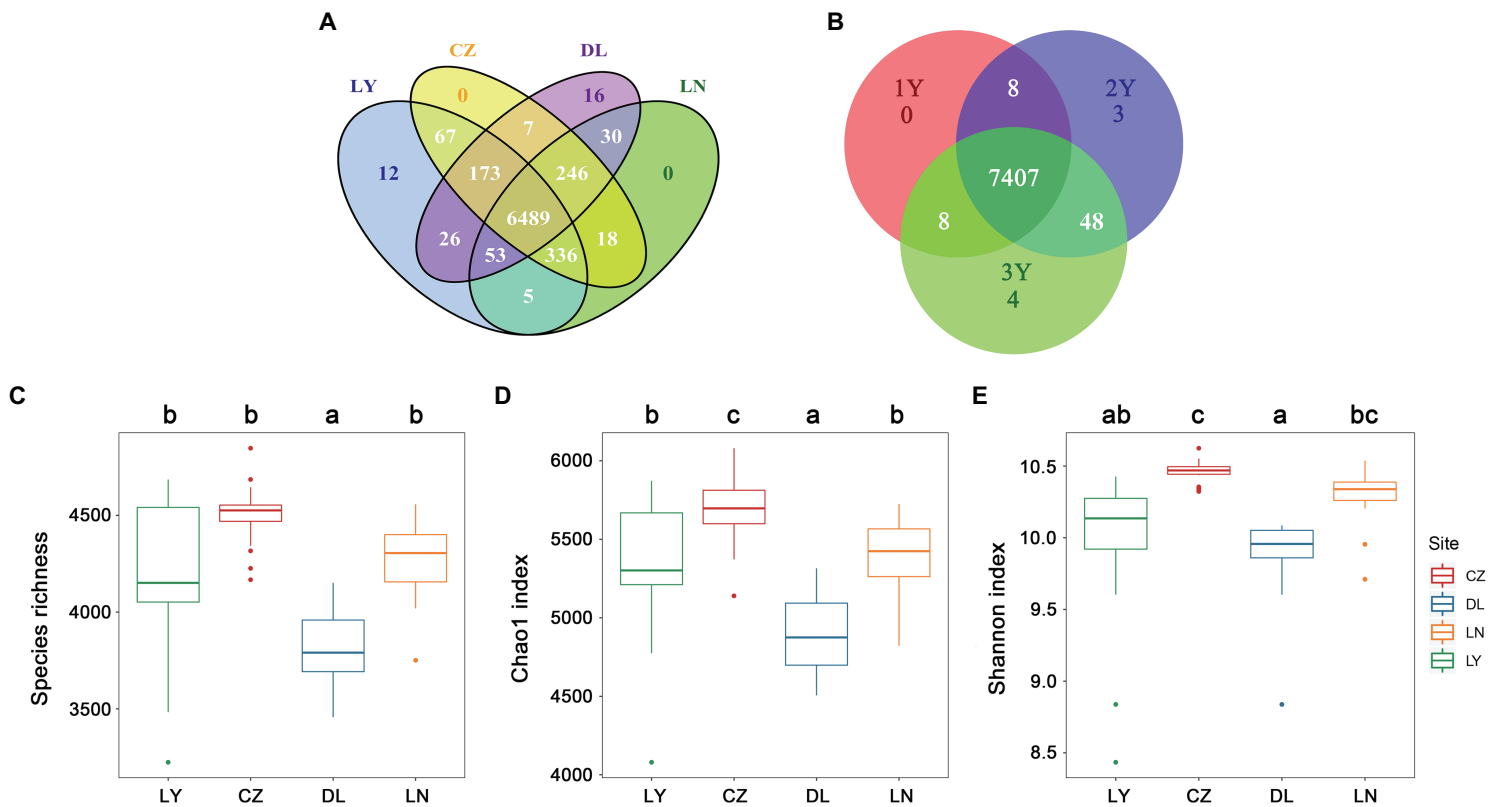
Diversity of the rhizosphere bacterial communities of *Sophora flavescens*. **(A,B)** The number of specific and shared operational taxonomic units (OTUs) of the rhizosphere bacterial communities from different geographic locations **(A)** or between different plant ages **(B)**. **(C–E)** The alpha diversity, including species richness **(C)**, Chao1 index **(D)**, and Shannon index **(E)**, of the bacterial communities in the rhizosphere samples from different geographic locations. Different lowercase letters indicate significant differences among geographical locations (*P* < 0.05). LN: Lingyuan; CZ: Changzhi; DL: Dali; LN: Luonan. 1Y: 1-year-old; 2Y: 2-year-old; 3Y: 3-year-old.

A total of 7,478 non-singleton OTUs were retrieved from 72 rhizosphere samples after normalization of the read numbers. The LY, CZ, DL, and LN samples shared a large proportion of OTUs (6,489 OTUs, 86.77%; Figure 1A). Moreover, 7,407 OTUs (99.05%) were shared by rhizosphere samples of *S. flavescens* of three ages (Figure 1B). The rhizosphere bacterial communities were less diverse (*P* < 0.05) in the DL samples (species richness: 3,817 ± 195; Chao1: 4,894.81 ± 263.57) than in the LY (species richness: 4,203 ± 401; Chao1: 5,330.36 ± 424.56), CZ (species richness: 4,503 ± 163; Chao1: 5,680.92 ± 211.38), and LN (species richness: 4,262 ± 197; Chao1: 5,382.46 ± 243.59) samples (Figures 1C,D). The Chao1 index was highest in the CZ samples (Figure 1D). Moreover, the Shannon index in the CZ samples (10.46 ± 0.08) was higher than that in the LY (9.97 ± 0.53) and DL (9.88 ± 0.29) samples (Figure 1E). No significant differences in the rhizosphere bacterial diversity were found among the three plant ages (Supplementary Figure S2). The Spearman’s rank correlation test revealed that the rhizosphere bacterial diversity was positively correlated with the MAP and the contents of soil OM and AN, but negatively correlated with the MAT, soil pH, and AK content (Supplementary Table S4).

**Figure 2:**
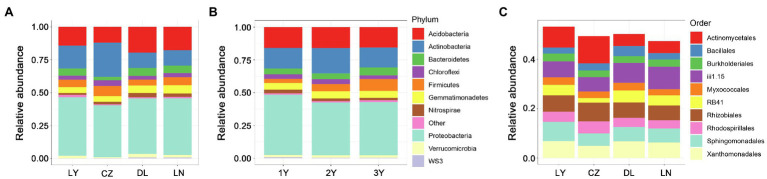
Taxonomic structure of the bacterial communities in the rhizosphere of *S. flavescens* from different geographic locations **(A,C)** or of different ages **(B)** at the phylum **(A,B)** or order **(C)** level. LN: Lingyuan; CZ: Changzhi; DL: Dali; LN: Luonan. 1Y: 1-year-old; 2Y: 2-year-old; 3Y: 3-year-old.

At the phylum level, Proteobacteria dominated the rhizosphere bacterial communities, followed by Actinobacteria and Acidobacteria (Figures 2A,B). These three most abundant phyla accounted for a large proportion (more than 70%) of the relative abundance of the rhizosphere bacterial communities. Compared with the LY, DL, and LN samples, the CZ samples hosted a higher relative abundance of Actinobacteria (26.07%) but a lower relative abundance of Proteobacteria (39.39%) and Acidobacteria (11.90%). The LY and DL samples presented the highest relative abundances of Proteobacteria (44.49%) and Acidobacteria (19.57%), respectively. We further characterized the rhizosphere bacterial communities at the order level. The results showed that the top 10 most abundant orders included six orders (Burkholderiales, Myxococcales, Rhizobiales, Rhodospirillales, Sphingomonadales, and Xanthomonadales) of the phylum Proteobacteria, two unidentified orders (iii1.15 and RB41) of the phylum Acidobacteria, one order (Actinomycetales) belonging to Actinobacteria and one order (Bacillales) belonging to Firmicutes (Figure 2C). We found that Actinomycetales dominated the rhizosphere bacterial communities in the CZ and LY samples, whereas the unidentified order iii1.15 (belonging to the phylum Acidobacteria) dominated the rhizosphere compartment in the DL and LN samples. Furthermore, Actinomycetales was more abundant in the CZ (11.03%) and LY (8.41%) samples than in the DL (4.85%) and LN (4.82%) samples. Conversely, the total relative abundance of the two orders of the phylum Actinobacteria in the CZ samples (7.76%) was lower than that in the LY (10.72%), DL (12.95%), and LN (13.15%) samples.

### Ecological Factors Shaping the Rhizosphere Bacterial Communities

**Figure 3:**
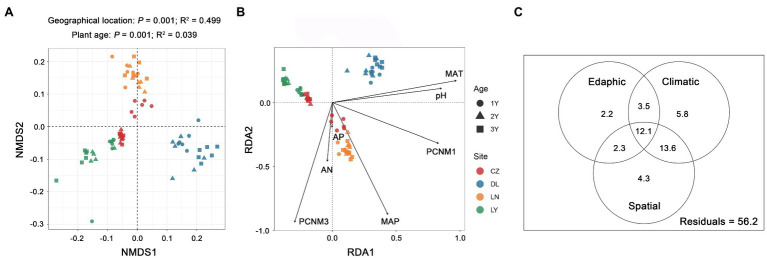
Variation in the rhizosphere bacterial communities of *S. flavescens*. **(A)** Nonmetric multidimensional scaling (NMDS) ordination of the rhizosphere bacterial communities based on the Bray-Curtis distance matrix. **(B)** Redundancy analysis (RDA) revealing significant spatial, climatic, and edaphic variables shaping the rhizosphere bacterial communities. **(C)** Variation partitioning analysis (VPA) indicating the independent and interactive impacts (the percentage of variation explained) of the spatial, climatic, and edaphic factors on the rhizosphere bacterial communities. LN: Lingyuan; CZ: Changzhi; DL: Dali; LN: Luonan. 1Y: 1-year-old; 2Y: 2-year-old; 3Y: 3-year-old. PCNM: principal coordinates of neighbor matrices; MAT: mean annual temperature; MAP: mean annual precipitation; AN: available N; AP: available P.

NMDS ordination plots based on the Bray–Curtis dissimilarity matrix displayed separated clustering of rhizosphere bacterial communities across the geographical locations and plant ages (Figure 3A). Similarly, PERMANOVA confirmed that both geographical location (*P* = 0.001) and plant age (*P* = 0.001) were significant driving factors for the variation in the rhizosphere bacterial community composition. However, geographical location (R^2^ = 0.499) had a more pivotal effect than plant age (R^2^ = 0.039). The results of RDA showed that two spatial variables (PCNM 1 and 3), two climatic variables (MAT and MAP), and three edaphic variables (pH, AN and AP) were significant drivers (*P* < 0.05) shaping the rhizosphere bacterial communities (Figure 3B). Among these ecological factors, the MAT (R^2^ = 0.185) was the strongest. The results of VPA revealed that the climatic factors (35.0%) explained a larger proportion of variance in the rhizosphere bacterial community composition than the spatial (32.3%) and edaphic (20.1%) factors (Figure 3C).

### Rhizosphere Bacteria Associated With Alkaloid Accumulation in Roots

**Figure 4:**
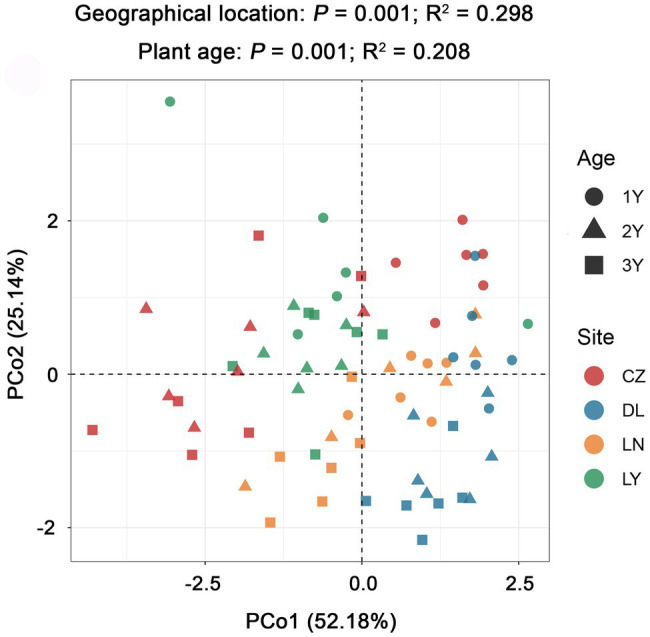
Variation in the root alkaloid accumulation of *S. flavescens* performed by principal coordinate analysis (PCoA) based on the Bray-Curtis distance matrix. LN: Lingyuan; CZ: Changzhi; DL: Dali; LN: Luonan. 1Y: 1-year-old; 2Y: 2-year-old; 3Y: 3-year-old.

PCoA grouped with PERMANOVA revealed significant differences in alkaloid accumulation across the geographical locations (*P* = 0.001; R^2^ = 0.298) and plant ages (*P* = 0.001; R^2^ = 0.208; Figure 4). By using the Mantel test, we found that the rhizosphere bacterial microbiota of *S. flavescens* was remarkably correlated with the contents of oxymatrine (*P* = 0.001; *R* = 0.230), sophoridine (*P* = 0.001; *R* = 0.351), and matrine + oxymatrine (*P* = 0.001; *R* = 0.226) in roots (Supplementary Table S5). Considering that the total content of matrine and oxymatrine in the roots of *S. flavescens* is used as a quality control marker for this medicinal plant in the Pharmacopoeia of the People’s Republic of China (Chinese Pharmacopoeia Commission, 2020), we focused on the association between the rhizosphere bacteria and the total content of these two molecules.

**Figure 5:**
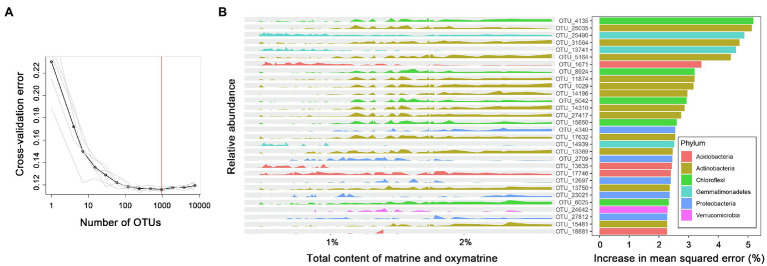
The top 30 important bacterial markers associated with the total accumulation of matrine and oxymatrine in the roots of *S. flavescens* based on a random forest model. **(A)** The 10-fold cross-validation with five repeats generates 935 bacterial marker OTUs correlated with the total content of matrine and oxymatrine. **(B)** The peak map plotted according to the relative abundance of the top 30 marker OTUs against the total content of matrine and oxymatrine.

A random forest machine learning algorithm was used to establish a model for the association. The 10-fold cross-validation with five repeats generated 935 important bacterial OTUs correlated with the total accumulation of matrine and oxymatrine in roots (R^2^ = 0.592; Figure 5A). In order of importance, the top 30 bacterial OTUs are exhibited in Figure 5B according to their relative abundance distribution in roots with different accumulations of matrine + oxymatrine. Within these 30 bacterial markers, OTU 4,135, belonging to Chloroflexi, contributed most to the total accumulation of matrine and oxymatrine. Taxa of the phylum Actinobacteria (10 OTUs, in expectation of OTUs 25,035 and 17,632) dominated the bacteria whose increase in relative abundance favored the total accumulation of matrine and oxymatrine. In contrast, the increase in the relative abundance of certain bacteria presented a negative influence on the total accumulation of matrine and oxymatrine, which included three taxa (OTUs 25,490, 13,741 and 14,939) of the phylum Gemmatimonadetes and two taxa (OTUs 1,671 and 13,635) of the phylum Acidobacteria. Additionally, for some bacteria, e.g., OTUs 2,709 and 12,697 (both of which belong to the Proteobacteria phylum), the association of their relative abundances with the total accumulation of matrine and oxymatrine could not be simply defined (Figure 5).

### Interactions Between Spatial, Climatic, and Edaphic Factors, Bacterial Attributes, and Alkaloid Accumulation

**Figure 6:**
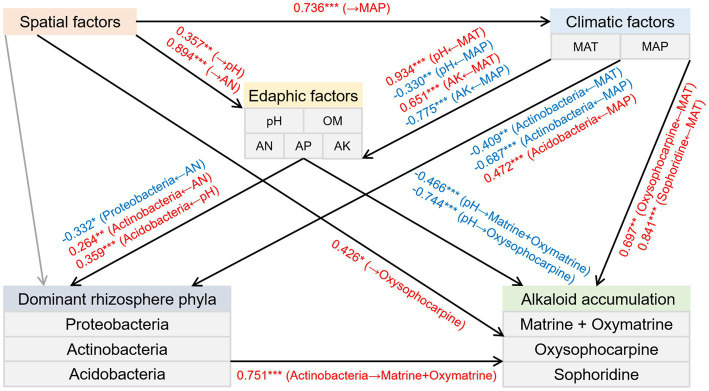
Structural equation model depicting the direct and indirect effects of the spatial, climatic, and edaphic factors and the relative abundance of three preponderant rhizosphere phyla on the accumulation of alkaloids in the roots of *S. flavescens* (*P* = 0.385; root-mean-squared error of approximation = 0.027; comparative fit index = 1.000). Black arrow lines: significant correlations; Gray arrow lines: correlations that are not significant; Red arrows: positive correlations; Blue arrows: negative correlations. Numbers between parameter boxes are indicative of the correlations. *P < 0.05; **P < 0.01; ***P < 0.001. MAT: mean annual temperature; MAP: mean annual precipitation; OM: organic matter; AN: available N; AP: available P; AK: available K.

SEM was used to estimate the direct and indirect associations regarding causation between the spatial, climatic, and edaphic factors, the relative abundance of three dominant rhizosphere phyla, and the accumulation of alkaloids. The results indicated the following (Figure 6): (1) The spatial factors directly influenced the MAP. (2) Soil pH and AK were positively affected by the MAT but negatively affected by the MAP. In addition, soil pH and AN were positively associated with the spatial factors. (3) The climatic factors had positive and negative effects on the relative abundance of Acidobacteria and Actinobacteria, respectively. Besides, the soil AN had positive and negative impacts on the relative abundance of Actinobacteria and Proteobacteria, respectively. In addition, the relative abundance of Acidobacteria was positively influenced by the soil pH. (4) Actinobacteria positively affected the total content of matrine and oxymatrine. Furthermore, the contents of matrine + oxymatrine and oxysophocarpine were negatively influenced by the soil pH. Moreover, the oxysophocarpine content was positively associated with the spatial factors and the MAT. (5) The sophoridine content was also positively impacted by the MAT.

## Discussion

Alkaloids, one of the major classes of plant SMs, are critical bioactive constituents in the medicinal plant *S. flavescens* (He et al., 2015). Although plant SMs can benefit plant fitness and human health, the accumulation of SMs can vary among individuals (Moore et al., 2014; Pang et al., 2021). In the present study, we also discovered variations in the accumulation of alkaloids among root samples of *S. flavescens*, with geographic location and plant age being important sources of variation (Figure 4). It is recognized that the intraspecific variation in plant SMs is not only dependent on plant genotype and ontogeny but also influenced by various environmental factors involving biotic and abiotic factors (Moore et al., 2014; Yang et al., 2018). The importance of plant-associated microbes, as vital biotic factors, for explaining plant SM polymorphisms in medicinal plants has been highlighted (Huang et al., 2018). In addition, plant rhizosphere is a hotspot for studying the impacts of plant-associated microbes on the accumulation of SMs in plants. Therefore, we determined the taxonomic structure of rhizosphere bacterial communities of *S. flavescens* and addressed the association between the rhizosphere bacterial microbiota and the accumulation of alkaloids in *S. flavescens*.

Plants host a taxonomically diverse microbiota but only a few dominant taxa (Müller et al., 2016). The dominance of the bacterial phyla Proteobacteria, Actinobacteria, Bacteroidetes, Acidobacteria, and Firmicutes was discovered in previous studies (Bulgarelli et al., 2012, 2015; Lundberg et al., 2012; Zarraonaindia et al., 2015; Fan et al., 2017; Zhang et al., 2019). In the present research, we found that the rhizosphere bacterial communities of the legume *S. flavescens* were dominated by Proteobacteria (mostly from the Rhizobiales order), followed by Actinobacteria, Acidobacteria, Firmicutes, Gemmatimonadetes, and Bacteroidetes (Figure 2), revealing similarly dominant phyla with previous studies.

Then, we explored the dissimilarities in the rhizosphere bacterial communities of *S. flavescens*. The results revealed distinct community compositions across the geographic locations and plant ages and indicated that the geographic location accounted for most of the variation (Figure 3A). Previous reports on maize and soybean plants also indicated that the largest proportion of variance in rhizosphere microbiota could be attributed to the geographic location (Peiffer et al., 2013; Zhang et al., 2021). The large spatial distance of sampling employed in the present study may result in the dominant contribution of geographic location in structuring the rhizosphere bacterial communities. The results of VPA showed that the spatial, climatic, and edaphic factors contributed to the variation in the rhizosphere bacterial community composition (Figure 3C), suggesting that dispersal limitation and environmental filtering could account for the turnover of the rhizosphere microbial communities of *S. flavescens* across the geographic locations (Zhang et al., 2018a, 2018b). We also conducted RDA ordination to identify the key spatial, climatic, and edaphic drivers responsible for the variation in the rhizosphere bacterial communities of *S. flavescens*. The results showed that two spatial variables (PCNM 1 and 3), two climatic variables (MAT and MAP), and three edaphic variables (pH, AN, and AP) were significant in driving bacterial community turnover (Figure 3B). Besides the geographic location, plant age could influence the assembly of rhizosphere microbiota (Micallef et al., 2009; Marques et al., 2014; Huang et al., 2020; Wang et al., 2020). Plants can select different microbes at the different stages of development, presumably for specific benefits (Chaparro et al., 2014). Overall, in the present study, the geographic location was a larger source of variation for shaping the rhizosphere bacterial communities than the plant age (Figure 3A). Similar results were obtained with grapevine plants (Manici et al., 2017).

We evaluated the correlation between the rhizosphere bacterial microbiota of *S. flavescens* and the accumulation of alkaloids in roots of the plant by using the Mantel test. The results elucidated significant correlations between the rhizosphere bacterial communities and the contents of oxymatrine, sophoridine, and matrine + oxymatrine (Supplementary Table S5). Accordingly, we used the random forest model to regress the relative abundance of the rhizosphere bacterial OTUs against the total content of matrine and oxymatrine to identify the potential beneficial bacteria for enhancing the total accumulation of these two molecules in roots of *S. flavescens*, which is used as a standard for the quality evaluation of this medicinal plant. We found that 59.2% of the variation in the matrine + oxymatrine content could be explained by the random forest model (Figure 5), which reveals the important influence of rhizosphere bacterial microbiota on the accumulation of these two molecules in the plant. Within the top 30 important bacteria, positive markers were mostly from Actinobacteria and Chloroflexi, while negative markers predominantly belonged to Gemmatimonadetes and Acidobacteria.

Actinobacteria members, ubiquitous in terrestrial habitats, are known to produce extensive SMs, many of which are of great importance for medicine and agriculture (Barka et al., 2016; Choudoir et al., 2019). Host plants establishing symbiotic associations with some members of Actinobacteria were reported to have improved resistance to biotic stresses through activation of defense pathways or upregulation of certain SMs (Conn et al., 2008; Kurth et al., 2014). In addition, Yan et al. (2014) found that *Streptomyces pactum* Act12, a member of Actinobacteria, could improve the growth of hairy roots of *Salvia miltiorrhiza* and promote the synthesis of tanshinone in hairy roots by upregulating the expression of corresponding biosynthetic genes. As shown in Figure 6, our SEM also indicated a positive and direct effect of Actinobacteria on the total accumulation of matrine and oxymatrine. Acidobacteria is also widespread and abundant in nearly all ecosystems (Kalam et al., 2020). However, owing to difficulties in cultivation, the phylum is rudimentarily explored (Kielak et al., 2016; Kalam et al., 2020). Most of the capabilities and ecological functions of Acidobacteria members, such as decomposition of various biopolymers and plant growth promotion, are predicted from their genomic and metagenomic information (Kalam et al., 2020). Although our knowledge in terms of their influence on plant SM accumulation was limited, the covariance analysis of our SEM revealed a negative correlation between the relative abundance of this phylum and the phylum Actinobacteria (*P* = 0.002; *R* = −0.525). This negative correlation could also be indicated by the Spearman’s rank correlation (*P* = 0.000; *R* = −0.778) and may allude to a competitive relationship between these two phyla, which may account for the negative association between the phylum Acidobacteria and the total accumulation of matrine and oxymatrine in the outcome of the random forest model (Figure 5). The phylum Chloroflexi has been found in anaerobic habitats, such as sediments and hot springs, exerting a role in biogeochemical carbon and nitrogen cycling (Hug et al., 2013; Speirs et al., 2019; Spieck et al., 2020). A previous study by Hu et al. (2018) demonstrated that Chloroflexi bacteria were attracted by root exudates of maize and were involved in host-medicated rhizosphere microbiota assembly to promote the growth and defense capability of the host. The phylum Gemmatimonadetes, containing phototrophic species, is also a nearly unexplored bacterial group (Zeng et al., 2020). The impacts of these microbes on the accumulation of bioactive components in *S. flavescens* should be illustrated in further studies based on isolation of the identified bacteria and cocultivation of them with the host plant.

Variations in abiotic factors, such as temperature, light, nutrients, and water, could also cause changes in the type and amount of plant SMs, which may be due to the regulation of the biosynthesis of SMs or a consequence of passive dilution or concentration due to the altered plant growth rate (Moore et al., 2014; Isah, 2019). Therefore, we applied SEM to evaluate the direct and indirect effects of the spatial, climatic, and edaphic factors and the relative abundance of the three dominant rhizosphere phyla on the accumulation of alkaloids in the roots of *S. flavescens*. The results showed that the spatial, climatic, and edaphic factors had both direct and indirect effects on the accumulation of alkaloids (Figure 6). The direct and indirect (through alteration of the relative abundance of Actinobacteria) effects of edaphic factors on the accumulation of alkaloids implies the impact of comprehensive soil-microbe-root interactions in the rhizosphere on bioactive component accumulation in medicinal plants.

In conclusion, the present study indicated that the accumulation of alkaloids in *S. flavescens* could be influenced by the rhizosphere bacterial communities of the plant. We identified the potentially beneficial bacteria associated with the total accumulation of matrine and oxymatrine with a random forest machine learning algorithm. In addition, the causal relationship among the spatial, climatic, and edaphic factors, the three most abundant rhizosphere phyla (Proteobacteria, Actinobacteria, and Acidobacteria) and the accumulation of alkaloids was indicated by SEM. We proposed rhizosphere engineering of specific microbial members for the control and improvement of the growth and quality of medicinal plants in the future.

## Data Availability Statement

The original contributions presented in the study are publicly available. This data can be found here: PRJNA780464 accession is not yet publicly available.

## Author Contributions

JChe and GY conceived the study. JChe and NL conducted the experiments, analyzed the data, and drafted the manuscript. JCha and KR participated in the experiments. JChe, GY, and JZ reviewed and edited the manuscript. All authors contributed to the article and approved the submitted version.

## Funding

This study was supported by the Plan of Shanxi Province Science and Technology Research (201901D211341), the Shanxi Higher Education Innovation Project (2019L0417), the Central Government Guides Local Scientific and Technological Development Fund Projects (YDZX 20201400001443), the Shanxi International Science and Technology Cooperation Project (201803D421065), the Science Research Start-up Fund for Doctor of Shanxi Province (SD1815), the Taiyuan City Science and Technology Project Special Talents Star Project (120247-08), and the Science Research Start-up Fund for Doctor of Shanxi Medical University (XD1816).

## Conflict of Interest

The authors declare that the research was conducted in the absence of any commercial or financial relationships that could be construed as a potential conflict of interest.

## Publisher’s Note

All claims expressed in this article are solely those of the authors and do not necessarily represent those of their affiliated organizations, or those of the publisher, the editors and the reviewers. Any product that may be evaluated in this article, or claim that may be made by its manufacturer, is not guaranteed or endorsed by the publisher.

## Footnotes

^1^ http://chelsa-climate.org/

## References

[ref1] Bao S. (2000). Soil and Agricultural Chemistry Analysis. Beijing: China Agriculture Press.

[ref2] Barka E. A. Vatsa P. Sanchez L. Gaveau-Vaillant N. Jacquard C. Klenk H. P.. (2016). Taxonomy, physiology, and natural products of *Actinobacteria*. Microbiol. Mol. Biol. Rev. 80, 1–43. doi: 10.1128/MMBR.00019-15, PMID: 26609051 PMC4711186

[ref3] Berendsen R. L. Pieterse C. M. J. Bakker P. A. H. M. (2012). The rhizosphere microbiome and plant health. Trends Plant Sci. 17, 478–486. doi: 10.1016/j.tplants.2012.04.001 22564542

[ref4] Borcard D. Gillet F. Legendre P. (2011). Numerical Ecology With R. New York: Springer Science and Business Media.

[ref5] Bulgarelli D. Garrido-Oter R. Münch P. C. Weiman A. Dröge J. Pan Y.. (2015). Structure and function of the bacterial root microbiota in wild and domesticated barley. Cell Host Microbe 17, 392–403. doi: 10.1016/j.chom.2015.01.011, PMID: 25732064 PMC4362959

[ref6] Bulgarelli D. Rott M. Schlaeppi K. van Themaat E. V. L. Ahmadinejad N. Assenza F.. (2012). Revealing structure and assembly cues for *Arabidopsis* root-inhabiting bacterial microbiota. Nature 488, 91–95. doi: 10.1038/nature11336, PMID: 22859207

[ref7] Caporaso J. G. Kuczynski J. Stombaugh J. Bittinger K. Bushman F. D. Costello E. K.. (2010). QIIME allows analysis of high-throughput community sequencing data. Nat. Methods 7, 335–336. doi: 10.1038/nmeth.f.303, PMID: 20383131 PMC3156573

[ref8] Caporaso J. G. Lauber C. L. Walters W. A. Berg-Lyons D. Huntley J. Fierer N.. (2012). Ultra-high-throughput microbial community analysis on the Illumina HiSeq and MiSeq platforms. ISME J. 6, 1621–1624. doi: 10.1038/ismej.2012.8, PMID: 22402401 PMC3400413

[ref9] Chaparro J. M. Badri D. V. Vivanco J. M. (2014). Rhizosphere microbiome assemblage is affected by plant development. ISME J. 8, 790–803. doi: 10.1038/ismej.2013.196, PMID: 24196324 PMC3960538

[ref10] Chen H. Wu H. Yan B. Zhao H. Liu F. Zhang H.. (2018). Core microbiome of medicinal plant *salvia miltiorrhiza* seed: a rich reservoir of beneficial microbes for secondary metabolism? Int. J. Mol. Sci. 19:672. doi: 10.3390/ijms19030672, PMID: 29495531 PMC5877533

[ref11] Chinese Pharmacopoeia Commission. (2020). The Pharmacopoeia of the People’s Republic of China, Beijing: China Medical Science Press.

[ref12] Choudoir M. Rossabi S. Gebert M. Helmig D. Fierer N. (2019). A phylogenetic and functional perspective on volatile organic compound production by *Actinobacteria*. mSystems 4, e00295–e00218. doi: 10.1128/mSystems.00295-18, PMID: 30863793 PMC6401417

[ref13] Conn V. M. Walker A. R. Franco C. M. M. (2008). Endophytic actinobacteria induce defense pathways in *Arabidopsis thaliana*. Mol. Plant Microbe Interact. 21, 208–218. doi: 10.1094/MPMI-21-2-0208, PMID: 18184065

[ref14] de Vries F. T. Griffiths R. I. Knight C. G. Nicolitch O. Williams A. (2020). Harnessing rhizosphere microbiomes for drought-resilient crop production. Science 368, 270–274. doi: 10.1126/science.aaz5192, PMID: 32299947

[ref15] Edgar R. C. (2010). Search and clustering orders of magnitude faster than BLAST. Bioinformatics 26, 2460–2461. doi: 10.1093/bioinformatics/btq461, PMID: 20709691

[ref16] Edgar R. C. Haas B. J. Clemente J. C. Quince C. Knight R. (2011). UCHIME improves sensitivity and speed of chimera detection. Bioinformatics 27, 2194–2200. doi: 10.1093/bioinformatics/btr381, PMID: 21700674 PMC3150044

[ref17] Edwards J. Johnson C. Santos-Medellín C. Lurie E. Podishetty N. K. Bhatnagar S.. (2015). Structure, variation, and assembly of the root-associated microbiomes of rice. Proc. Natl. Acad. Sci. U.S.A. 112, E911–E920. doi: 10.1073/pnas.1414592112, PMID: 25605935 PMC4345613

[ref18] Fan K. Cardona C. Li Y. Shi Y. Xiang X. Shen C.. (2017). Rhizosphere-associated bacterial network structure and spatial distribution differ significantly from bulk soil in wheat crop fields. Soil Biol. Biochem. 113, 275–284. doi: 10.1016/j.soilbio.2017.06.020

[ref19] Fan K. Delgado-Baquerizo M. Zhu Y. Chu H. (2020). Crop production correlates with soil multitrophic communities at the large spatial scale. Soil Biol. Biochem. 151:108047. doi: 10.1016/j.soilbio.2020.108047

[ref20] He X. Fang J. Huang L. Wang J. Huang X. (2015). *Sophora flavescens* Ait.: traditional usage, phytochemistry and pharmacology of an important traditional Chinese medicine. J. Ethnopharmacol. 172, 10–29. doi: 10.1016/j.jep.2015.06.010, PMID: 26087234

[ref21] Hu L. Robert C. A. M. Cadot S. Zhang X. Ye M. Li B.. (2018). Root exudate metabolites drive plant-soil feedbacks on growth and defense by shaping the rhizosphere microbiota. Nat. Commun. 9, 2738. doi: 10.1038/s41467-018-05122-7, PMID: 30013066 PMC6048113

[ref22] Huang R. Chen P. Wang X. Li H. Zuo L. Zhang Y.. (2020). Structural variability and niche differentiation of the rhizosphere and endosphere fungal microbiome of *Casuarina equisetifolia* at different ages. Braz. J. Microbiol. 51, 1873–1884. doi: 10.1007/s42770-020-00337-7, PMID: 32661898 PMC7688850

[ref23] Huang W. Long C. Lam E. (2018). Roles of plant-associated microbiota in traditional herbal medicine. Trends Plant Sci. 23, 559–562. doi: 10.1016/j.tplants.2018.05.003, PMID: 29802067

[ref24] Hug L. A. Castelle C. J. Wrighton K. C. Thomas B. C. Sharon I. Frischkorn K. R.. (2013). Community genomic analyses constrain the distribution of metabolic traits across the Chloroflexi phylum and indicate roles in sediment carbon cycling. Microbiome 1, 22. doi: 10.1186/2049-2618-1-22, PMID: 24450983 PMC3971608

[ref25] Isah T. (2019). Stress and defense responses in plant secondary metabolites production. Biol. Res. 52, 39. doi: 10.1186/s40659-019-0246-3, PMID: 31358053 PMC6661828

[ref26] Jin T. Wang Y. Huang Y. Xu J. Zhang P. Wang N.. (2017). Taxonomic structure and functional association of foxtail millet root microbiome. GigaScience 6, 1–12. doi: 10.1093/gigascience/gix089, PMID: 29050374 PMC7059795

[ref27] Kalam S. Basu A. Ahmad I. Sayyed R. Z. El-Enshasy H. A. Dailin D. J.. (2020). Recent understanding of soil Acidobacteria and their ecological significance: a critical review. Front. Microbiol. 11:580024. doi: 10.3389/fmicb.2020.580024, PMID: 33193209 PMC7661733

[ref28] Kembel S. W. O’Connor T. K. Arnold H. K. Hubbell S. P. Wright S. J. Green J. L. (2014). Relationships between phyllosphere bacterial communities and plant functional traits in a neotropical forest. Proc. Natl. Acad. Sci. U.S.A. 111, 13715–13720. doi: 10.1073/pnas.1216057111, PMID: 25225376 PMC4183302

[ref29] Kielak A. M. Barreto C. C. Kowalchuk G. A. van Veen J. A. Kuramae E. E. (2016). The ecology of Acidobacteria: moving beyond genes and genomes. Front. Microbiol. 7:744. doi: 10.3389/fmicb.2016.00744, PMID: 27303369 PMC4885859

[ref30] Kui L. Chen B. Chen J. Sharifi R. Dong Y. Zhang Z.. (2021). A comparative analysis on the structure and function of the *Panax notoginseng* rhizosphere microbiome. Front. Microbiol. 12:673512. doi: 10.3389/fmicb.2021.673512, PMID: 34177857 PMC8219928

[ref31] Kurth F. Mailänder S. Bönn M. Feldhahn L. Herrmann S. Große I.. (2014). *Streptomyces*-induced resistance against oak powdery mildew involves host plant responses in defense, photosynthesis, and secondary metabolism pathways. Mol. Plant Microbe Interact. 27, 891–900. doi: 10.1094/MPMI-10-13-0296-R, PMID: 24779643

[ref32] Lee S. M. Kong H. G. Song G. C. Ryu C. M. (2021). Disruption of Firmicutes and Actinobacteria abundance in tomato rhizosphere causes the incidence of bacterial wilt disease. ISME J. 15, 330–347. doi: 10.1038/s41396-020-00785-x, PMID: 33028974 PMC7852523

[ref33] Li Y. Kong D. Fu Y. Sussman M. R. Wu H. (2020). The effect of developmental and environmental factors on secondary metabolites in medicinal plants. Plant Physiol. Biochem. 148, 80–89. doi: 10.1016/j.plaphy.2020.01.006, PMID: 31951944

[ref34] Liu S. Wang Z. Niu J. Dang K. Zhang S. Wang S.. (2021). Changes in physicochemical properties, enzymatic activities, and the microbial community of soil significantly influence the continuous cropping of *Panax quinquefolius* L. (American ginseng). Plant Soil 463, 427–446. doi: 10.1007/s11104-021-04911-2

[ref35] Lundberg D. S. Lebeis S. L. Paredes S. H. Yourstone S. Gehring J. Malfatti S.. (2012). Defining the core *Arabidopsis thaliana* root microbiome. Nature 488, 86–90. doi: 10.1038/nature11237, PMID: 22859206 PMC4074413

[ref36] Manici L. M. Saccà M. L. Caputo F. Zanzotto A. Gardiman M. Fila G. (2017). Long-term grapevine cultivation and agro-environment affect rhizosphere microbiome rather than plant age. Appl. Soil Ecol. 119, 214–225. doi: 10.1016/j.apsoil.2017.06.027

[ref37] Marques J. M. da Silva T. F. Vollu R. E. Blank A. F. Ding G. C. Seldin L.. (2014). Plant age and genotype affect the bacterial community composition in the tuber rhizosphere of field-grown sweet potato plants. FEMS Microbiol. Ecol. 88, 424–435. doi: 10.1111/1574-6941.12313, PMID: 24597529

[ref38] Micallef S. A. Channer S. Shiaris M. P. Colón-Carmona A. (2009). Plant age and genotype impact the progression of bacterial community succession in the *Arabidopsis* rhizosphere. Plant Signal. Behav. 4, 777–780. doi: 10.4161/psb.4.8.9229, PMID: 19820328 PMC2801398

[ref39] Moore B. D. Andrew R. L. Külheim C. Foley W. J. (2014). Explaining intraspecific diversity in plant secondary metabolites in an ecological context. New Phytol. 201, 733–750. doi: 10.1111/nph.12526 24117919

[ref40] Müller D. B. Vogel C. Bai Y. Vorholt J. A. (2016). The plant microbiota: systems-level insights and perspectives. Annu. Rev. Genet. 50, 211–234. doi: 10.1146/annurev-genet-120215-034952, PMID: 27648643

[ref41] Na X. Ma S. Ma C. Liu Z. Xu P. Zhu H.. (2021). *Lycium barbarum* L. (goji berry) monocropping causes microbial diversity loss and induces *Fusarium* spp. enrichment at distinct soil layers. Appl. Soil Ecol. 168:104107. doi: 10.1016/j.apsoil.2021.104107

[ref42] Pang Z. Chen J. Wang T. Gao C. Li Z. Guo L.. (2021). Linking plant secondary metabolites and plant microbiomes: A review. Front. Plant Sci. 12:621276. doi: 10.3389/fpls.2021.621276, PMID: 33737943 PMC7961088

[ref43] Peiffer J. A. Spor A. Koren O. Jin Z. Tringe S. G. Dangl J. L.. (2013). Diversity and heritability of the maize rhizosphere microbiome under field conditions. Proc. Natl. Acad. Sci. U.S.A. 110, 6548–6553. doi: 10.1073/pnas.1302837110, PMID: 23576752 PMC3631645

[ref44] Peres-Neto P. R. Legendre P. Dray S. Borcard D. (2006). Variation partitioning of species data matrices: estimation and comparison of fractions. Ecology 87, 2614–2625. doi: 10.1890/0012-9658(2006)87, PMID: 17089669

[ref45] Quast C. Pruesse E. Yilmaz P. Gerken J. Schweer T. Yarza P.. (2013). The SILVA ribosomal RNA gene database project: improved data processing and web-based tools. Nucleic Acids Res. 41, D590–D596. doi: 10.1093/nar/gks1219, PMID: 23193283 PMC3531112

[ref46] R Development Core Team (2021). R: A Language and Environment for Statistical Computing. R Foundation for Statistical Computing. Available at: http://www.R-project.org/

[ref47] Song J. Han Y. Bai B. Jin S. He Q. Ren J. (2019). Diversity of arbuscular mycorrhizal fungi in rhizosphere soils of the Chinese medicinal herb *Sophora flavescens* Ait. Soil Tillage Res. 195:104423. doi: 10.1016/j.still.2019.104423

[ref48] Speirs L. B. M. Rice D. T. F. Petrovski S. Seviour R. J. (2019). The phylogeny, biodiversity, and ecology of the *Chloroflexi* in activated sludge. Front. Microbiol. 10:2015. doi: 10.3389/fmicb.2019.02015, PMID: 31572309 PMC6753630

[ref49] Spieck E. Spohn M. Wendt K. Bock E. Shively J. Frank J.. (2020). Extremophilic nitrite-oxidizing *Chloroflexi* from Yellowstone hot springs. ISME J. 14, 364–379. doi: 10.1038/s41396-019-0530-9, PMID: 31624340 PMC6976673

[ref50] Sun X. Yang Y. Liu T. Huang H. Kuang Y. Chen L. (2021). Evaluation of the wound healing potential of *Sophora alopecuroides* in SD rat's skin. J. Ethnopharmacol. 273:113998. doi: 10.1016/j.jep.2021.113998, PMID: 33689799

[ref52] Wang Q. Sun H. Li M. Xu C. Zhang Y. (2020). Different age-induced changes in rhizosphere microbial composition and function of *Panax ginseng* in transplantation mode. Front. Plant Sci. 11:563240. doi: 10.3389/fpls.2020.563240, PMID: 33281838 PMC7688891

[ref53] Yan Y. Zhang S. Yang D. Zhang J. Liang Z. (2014). Effects of *Streptomyces pactum* Act12 on *salvia miltiorrhiza* hairy root growth and tanshinone synthesis and its mechanisms. Appl. Biochem. Biotechnol. 173, 883–893. doi: 10.1007/s12010-014-0876-4, PMID: 24733528

[ref54] Yang L. Wen K. Ruan X. Zhao Y. Wei F. Wang Q. (2018). Response of plant secondary metabolites to environmental factors. Molecules 23, 762. doi: 10.3390/molecules23040762, PMID: 29584636 PMC6017249

[ref55] Zarraonaindia I. Owens S. M. Weisenhorn P. West K. Hampton-Marcell J. Lax S.. (2015). The soil microbiome influences grapevine-associated microbiota. mBio 6, e02527–e02514. doi: 10.1128/mBio.02527-14, PMID: 25805735 PMC4453523

[ref56] Zeng Y. Nupur N. W. Madsen A. M. Chen X. Gardiner A. T. Koblížek M. (2020). *Gemmatimonas groenlandica* sp. nov. is an aerobic anoxygenic phototroph in the phylum Gemmatimonadetes. Front. Microbiol. 11:606612. doi: 10.3389/fmicb.2020.606612, PMID: 33519753 PMC7844134

[ref57] Zhang J. Liu Y. Zhang N. Hu B. Jin T. Xu H.. (2019). *NRT1.1B* is associated with root microbiota composition and nitrogen use in field-grown rice. Nat. Biotechnol. 37, 676–684. doi: 10.1038/s41587-019-0104-4, PMID: 31036930

[ref58] Zhang R. Vivanco J. M. Shen Q. (2017). The unseen rhizosphere root-soil-microbe interactions for crop production. Curr. Opin. Microbiol. 37, 8–14. doi: 10.1016/j.mib.2017.03.008, PMID: 28433932

[ref59] Zhang J. Xing P. Niu M. Wei G. Shi P. (2021). Taxonomic compositions and co-occurrence relationships of protists in bulk soil and rhizosphere of soybean fields in different regions of China. Front. Microbiol. 12:738129. doi: 10.3389/fmicb.2021.73812, PMID: 34603268 PMC8485050

[ref60] Zhang B. Zhang J. Liu Y. Guo Y. Shi P. Wei G. (2018a). Biogeography and ecological processes affecting root-associated bacterial communities in soybean fields across China. Sci. Total Environ. 627, 20–27. doi: 10.1016/j.scitotenv.2018.01.230, PMID: 29426141

[ref61] Zhang J. Zhang B. Liu Y. Guo Y. Shi P. Wei G. (2018b). Distinct large-scale biogeographic patterns of fungal communities in bulk soil and soybean rhizosphere in China. Sci. Total Environ. 644, 791–800. doi: 10.1016/j.scitotenv.2018.07.016, PMID: 29990927

[ref62] Zhang J. Zhang N. Liu Y. Zhang X. Hu B. Qin Y.. (2018c). Root microbiota shift in rice correlates with resident time in the field and developmental stage. Sci. China Life Sci. 61, 613–621. doi: 10.1007/s11427-018-9284-4, PMID: 29582350

