# Supplementary material for: Taxonomic Structure of Rhizosphere Bacterial Communities and Its Association With the Accumulation of Alkaloidal Metabolites in Sophora flavescens
Source: Front Microbiol. 2021 Dec 14;12:781316. doi: 10.3389/fmicb.2021.781316 (PMC8712762; doi:10.3389/fmicb.2021.781316)
Supplement: Supplementary file 1 [file Data_Sheet_1.docx]

**Supplemental Materials**





Fig. S1 Chromatograms of a mixture of oxymatrine, oxysophocarpine, sophoridine and matrine (black) and *S*. *flavescens* samples (red). 1, oxymatrine; 2, oxysophocarpine; 3, sophoridine; 4, matrine.


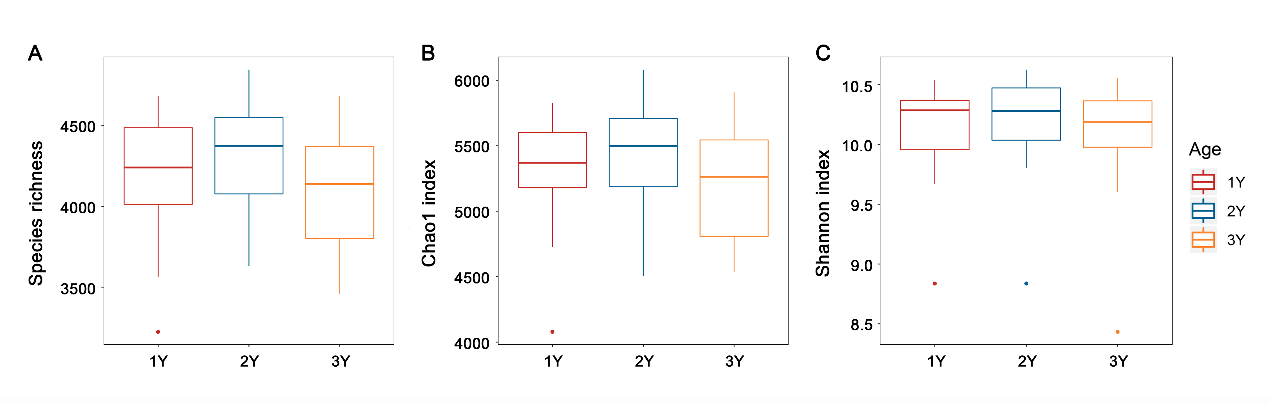


Fig. S2 The alpha diversity, including species richness (A), Chao1 index (B) and Shannon index (C), of the bacterial communities in the rhizosphere of *Sophora* *flavescens* of different ages. 1Y: 1-year-old; 2Y: 2-year-old; 3Y: 3-year-old.

Table S1 Information on the sampling sites of *Sophora* *flavescens*.

| Field NO. | Location | Longitude | Latitude | Plant age | Replicate |
| --- | --- | --- | --- | --- | --- |
| 1 | Linyuan, Liaoning | 119°14′45″ | 41°11′51″ | 1 | 6 |
| 2 | Linyuan, Liaoning | 119°15′3″ | 41°11′8″ | 2 | 6 |
| 3 | Linyuan, Liaoning | 119°14′48″ | 41°11′57″ | 3 | 6 |
| 4 | Changzhi, Shanxi | 113°1′13″ | 36°2′3″ | 1 | 6 |
| 5 | Changzhi, Shanxi | 113°1′11″ | 36°2′2″ | 2 | 6 |
| 6 | Changzhi, Shanxi | 113°1′17″ | 36°1′54″ | 3 | 6 |
| 7 | Dali, Shaanxi | 110°12′16″ | 34°43′1″ | 1 | 6 |
| 8 | Dali, Shaanxi | 110°11′41″ | 34°42′56″ | 2 | 6 |
| 9 | Dali, Shaanxi | 110°11′42″ | 34°42′56″ | 3 | 6 |
| 10 | Luonan, Shaanxi | 113°7′39″ | 34°15′42″ | 1 | 6 |
| 11 | Luonan, Shaanxi | 113°7′42″ | 34°15′49″ | 2 | 6 |
| 12 | Luonan, Shaanxi | 113°7′42″ | 34°15′47″ | 3 | 6 |

Table S2 Spatial, climatic and soil physiochemical parameters.

Table S3 Contents of alkaloids in roots of *S*. *flavescens*.

Table S4 Spearman's rank correlations between the environmental factors and bacterial diversity (Shannon index) in the rhizosphere of *S*. *flavescens*.

| Environmental factors | *P* value | *R* | Environmental factors | *P* value | *R* |
| --- | --- | --- | --- | --- | --- |
| MAP | 0.000 | 0.496 | MAT | 0.021 | –0.271 |
| Soil pH | 0.021 | –0.272 | OM content | 0.000 | 0.611 |
| AN content | 0.000 | 0.542 | AP content | 0.063 | 0.220 |
| AK content | 0.000 | –0.509 |  |  |  |

MAT: mean annual temperature; MAP: mean annual precipitation; OM: organic matter; AN: available N; AP: available P; AK: available K.

Table S5 Mantel test for correlation between the rhizosphere bacterial microbiota and the accumulation of alkaloids in the roots of *S*. *flavescens*.

| Alkaloids | *P* value | *R* |
| --- | --- | --- |
| Total alkaloids | 0.905 | –0.051 |
| Oxymatrine | 0.001 | 0.230 |
| Oxysophocarpine | 0.816 | –0.036 |
| Sophoridine | 0.001 | 0.351 |
| Matrine | 0.417 | 0.008 |
| Matrine + Oxymatrine | 0.001 | 0.226 |
